# How Management Practices Within a Poultry House During Successive Flock Rotations Change the Structure of the Soil Microbiome

**DOI:** 10.3389/fmicb.2019.02100

**Published:** 2019-09-13

**Authors:** Tawni L. Crippen, Cynthia L. Sheffield, Baneshwar Singh, J. Allen Byrd, Ross C. Beier

**Affiliations:** ^1^Southern Plains Agricultural Research Center, Agricultural Research Service, United States Department of Agriculture, College Station, TX, United States; ^2^Department of Forensic Science, Virginia Commonwealth University, Richmond, VA, United States

**Keywords:** poultry production, microbial community, management practices, microbiome, soil

## Abstract

The microbiome within a poultry production house influences the attainment of physiologically strong birds and thus food safety and public health. Yet little is known about the microbial communities within the house and the effects on the soil microbes onto which the houses are placed; nor the effects of management practices on their equilibrium. This study looked at the soil bacterial microbiome before a broiler house was constructed, then through 11 flock rotations (2.5 years) that included a partial clean-out and a total clean-out within the management regimen. Major shifts were observed, occurring at the taxonomic class level, related to the introduction of bedding and birds on top of the soil. The partial clean-out of litter did not change the soil bacterial community in any substantial way, only prompting a temporary increase in some genera; however, the total litter clean-out caused a major increase in a cohort of Actinobacteria. The underlying soil contained bacteria beneficial for poultry metabolism, such as *Lactobacillus*, *Faecalibacterium*, *Bacteriodes*, and *Ruminococcus*. Additionally, management practices affected the class structure of the soil bacterial community beneath the poultry house. The scheduling of these practices should be leveraged to exploit maintenance of beneficial bacteria that maximize microbiome contributions to bird production processes, while minimizing possible antibiotic-resistant bacteria and environmental effects.

## Introduction

Global production of broiler meat is around 92 million metric tons. The United States leads the world in broiler meat production, for both domestic consumption and international export producing about 19 million metric tons in 2018 (USDA, 2018a, b). The growth in the global demand for poultry meat has resulted in the expansion of concentrated animal feeding operations (CAFO), but to manage such an increase in animal density at facilities, a strategy for animal wellbeing and disease prevention and control is paramount (Godfray et al., 2010; Mathews and Haley, 2014). The industry must optimize nutrition and select for animals with a balance of high performance, fast growth, maximum yield, efficient feed conversion rates and strong physiological functionality to decreased susceptibility to structural issues and disease (Fadiel et al., 2005). Additionally, such facilities must manage the wastes produced by the operations to reduce ecological damage.

The microbiome of the various elements within the poultry house plays a part in all these management issues. Yet little is known about the microbial communities, their constituents, their functions or the effects of various management practices on their equilibrium. The influence of the poultry on the soil where a dirt house floor type poultry house is built is of special interest. According to Bakker et al. (2018) broad rearrangements in bulk soil community structure can be caused by various agricultural management styles. In other words, it is vital that we understand how soil microbiomes react to the influences of agricultural management practices in order to ensure soil health. Shange et al. (2013) examined microbiomes of soil under poultry houses, in adjacent pastures and under stored litter; and observed sizeable alterations in the specific taxa abundance between these environments. They also observed a greater abundance of pathogenic bacteria under the poultry houses which suggests a possible threat of continual recontamination of new bedding materials and birds. Therefore, studies evaluating the composition of the microbiome are of fundamental importance to any animal production operation as first step to determine how to integrate and maximize their contribution to the production processes while minimizing any long-term negative environmental effects.

This research was designed to determine the microbiome within the soil beneath a newly constructed dirt-floored broiler house over a two and a half year period, from initial site selection and application of the pad dirt, through 11 consecutive flock rotations. The study also integrated how management practices affect retention or decline of different bacteria that comprise the soil environment.

## Materials and Methods

### Site Description

Several new dirt-floored broiler production houses were constructed on an open range Post Oak Savannah greater than 1 mile from the nearest pre-existing broiler production facility in NW Robertson County, TX, United States (Gould et al., 1960). The soil was a fine, smectitic thermic Udertic Paleustalfs with slopes ranging from zero to three percent. The research was conducted from February 2008 to August 2010. The broiler facility was a standard tunnel ventilated metal house, 14 m wide (North/South) by 152.4 m in length (East/West) in size placed on a 25 cm thick Pad of commercial grade clay-based topsoil (Pad). Alternating water and feed lines ran the entire length of the house spaced at 1.52, 2.44, 3.66, 4.57, and 6.10 m from the South wall. Over the research period 11 flocks were grown out in the facility, over an average duration of 59 ± 6 days, and the house was left unoccupied for an average of 11 ± 5 days between flock rotations. Government permits and approvals for the work were not needed, as the land was privately owned, and we had permission to conduct this research from the landowner. Full personal protective equipment and sterile technique was used for personnel and equipment throughout the collection and no other sites were visited on the same day as collection from this site.

### Management Practices

For each flock rotation the birds were placed in the house at 1 day of age and confined to half of the house for a 2 weeks period, then allowed access to the entire house. The chickens were reared from 1–2 days of age through 6–9 weeks of age. Each flock had a stocking density of one broiler per 0.1 m^2^ (25,800 birds per rotation). Approximately 32 MT of fresh pine chip bedding (PCB) (15.3 cm) was added to the floor of the house prior to the arrival of the first flock. The Ross^®^ 708 broiler chicken was stocked and fed a corn/soy-based ration (see regime in Supplementary Table S1). After the 7th flock rotation, the producer performed a partial house clean-out (PCO), consisting of the removal of the top caked layer of hardened manure and 5–8 cm of litter. Litter is defined as bedding after use by the birds; and consists of bedding, chicken manure, urine, carrion, feathers, insects, spilled feed, leaked water, or any other materials deposited over the grow-out period. Fresh PCB (6.4 cm) was then added to the house. A total house clean-out (TCO) was performed after the 9th flock rotation consisting of removal of all litter plus 1–3 cm of the Pad-soil. Fresh PCB (15.3 cm) was added to the house prior to the 10th flock.

### Poultry House Collections

To assure the entire area was sampled, prior to groundbreaking for the new construction, the location of the house was plotted into 27 sectors (16.9 × 4.7 m) the length and width of the proposed house site, and the top 7.6 cm of native soil was sampled by taking a sample from within each sector then pooling into a composite sample for analysis (Native). After addition of 25.4 cm of Pad soil to the site, the top 7.6 cm of soil was again collected from within the 27 sectors the length and width of the impending house footprint prior to the construction of the broiler house and combined into a composite sample for analysis (Pad). After construction of the poultry house, approximately 15.3 cm of PCB was added throughout the house followed by placement of the birds. After placement of bedding and birds, the top 7.6 cm of Pad soil was collected by scraping away the litter to access the soil beneath. Logistical issues with the producer resulted in the following sampling regimes for the flocks: For Flock 1 and 2 soil was collected five times, on alternate weeks during the grow-out period with the first collection made on the first day of bird placement and last collection within 1 day of bird removal; Flock 3 – no collections; Flock 4 through 11- soil was collected on day 1 after bird placement and again within 1 day of bird removal.

For collection purposes, the house was divided into Side A and Side B because for the first 2 weeks of each flock rotation the birds were restricted to Side B by a brooder fence. Replicate samples were collected from each side using three feed and three water lines as boundaries at the interval length of five feeder pan stations (approximately 13.5 m) with the starting feeder randomly assigned. Three samples were taken per line, per house side (A or B) within 1 m of a line at these intervals. The 4th water line was not sampled. Soil was collected using a 12.7 cm long spade, which was thoroughly cleaned with Clorox^®^ wipes between sample sites. Each sample was made by scraping away the layer of litter until soil was exposed; the sample was taken and placed into an individual sterile zip-top bag. The total number of soil samples collected over the course of the study was 936. Samples were transported from the field site on ice packs and kept at 4°C until extraction. The samples for side-A line (feed or water) and for side-B line (feed or water) were combined into composite samples for each time point.

### Metagenomic Analyses

#### Sequencing

DNA was extracted using a FastDNA^TM^ SPIN Kit for Soil (MP Biomedicals, Santa Ana, CA, United States) per manufacturer instructions. Samples were sent to the Research and Testing Laboratory for 16S rRNA 454-pyrosequencing^1^ using the universal bacterial primer pair 27F (5′-GAGTTTG ATCNTGGCTCAG) and 519R (5′-GTNTTACNGCGGCKG CTG) by bacterial tag-encoded FLX-Titanium pyrosequencing (bTEFAP) method (Dowd et al., 2010) in Genome Sequencer FLX System (Roche, Nutley, NJ, United States). All FLX related procedures were performed following Genome Sequencer FLX System manufacturers instructions (Roche, Nutley, NJ, United States).

#### Data Analyses

Sequencing errors from all sequences were minimized using program PyroNoise (Quince et al., 2011) as implemented in Mothur v 1.35 (Schloss et al., 2009). Low quality regions of the sequences were trimmed using the sliding window (50 bp; Q35) option in Mothur v 1.35. Sequence with homopolymer length >8 bp, and total length <200 bp were removed from further analyses, and the rest were checked for chimera formation using program Uchime (Edgar et al., 2011) and using the most abundant sequence as reference data, as implemented in Mothur v 1.35.1. Suspected chimeric sequences were deleted and the rest good quality sequences (111954) were utilized for hierarchical classification using Naïve Bayesian rRNA classifier version 2.2 (Wang et al., 2007) as implemented in Mothur v 1.35.1. Only sequences having ≥80% bootstrap support were considered classified at a particular hierarchical level. To avoid spurious OTU count because of the different number of sequence reads in different samples, all sequences were rarefied (subsampled) to 463 reads and α-diversity indices (Inverse Simpson and Shannon) were calculated from OTU at 3% and 10% genetic distances. Yue and Clayton measure of dissimilarity was calculated from OTU at 3% genetic distance. Phylogeny based β-diversity (weighted and unweighted Unifrac) distances were also calculated for all samples. Sub-sampling caused a loss of nine samples from further analysis. Yue and Clayton distances were utilized for non-metric multidimensional scaling (NMDS) plot in Mothur v 1.35.1. NMDS data from first three axes for all treatments (Native, Pad, and Flocks) were plotted using the rgl package in R version 3.1.0 (R Development.Core.Team., 2011).

Heat maps graphics were generated for all classified class and genera from different treatments using natural log transformed percent relative sequence abundance profiles in the gplots package of R version 3.0.1. The 0% values were converted into 0.01% for log transformation. Heat map graphic for class changes (Figure 4) was generated in Prism 7 (Graph Pad, La Jolla, CA, United States). Native, Pad and Flocks on the *X*-axis were clustered based on weighted and unweighted Unifrac distances. All trees were edited using FigTree v1.3.1^2^. All raw sequence files were submitted to European Nucleotide Archive Database as part of the study PRJEB29406 (accession # ERS2859789–ERS2859880).

### Statistical Analysis

Initially analyses were performed for the poultry house collection by analysis of molecular variance (*P* < 0.0001). Results demonstrated no significant differences between water versus feed line collection sites, or Side A versus Side B collection sites. Therefore, these samples were combined based on Flock rotation for further analysis. After removal of nine samples due to low sequence numbers the total samples made from soil collected from each flock rotation was *n* = 1, 1, 5, 20, 8, 8, 5, 8, 5, 6, 8, 8 for Native, Pad, Flocks 1, 2 and Flocks 4–11, respectively.

## Results

The environment within broiler production houses are controlled to rear chickens from 1–2 days of age through 6–9 weeks of age. The house used in this study averaged 59 days of rearing and 11 days between flock rotations. Chickens are hunt-and-peck, coprophagous feeders, that frequently contact all aspects of the house environment from soil to litter, feeders, waterers, and invading insects. To investigate management practice effects on the soil microbial community within the broiler rearing house, samples collected were timed to flock rotations and clean-outs.

**FIGURE 1:**
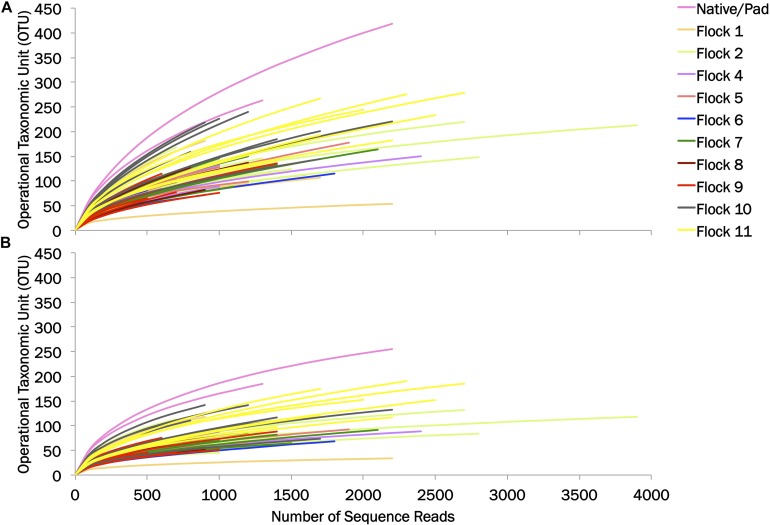
Rarefaction curves of operational taxonomic units (OTU) at **(A)** 0.03 and **(B)** 0.10 genetic distances.

The diversity of the bacterial genera in the soil between the Native and Pad and successive flock rotations was determined at 0.03 and 0.10 genetic distances (Table 1). Rarefaction curves are shown in Figure 1. Diversity indices provide information about the rarity and commonness of species present in the community structure. The Inverse Simpson Index showed a stark decrease in diversity of genera following application of bedding and the introduction of birds (Flock 1). Using the Simpsons index which considers both the richness (the number of unique species present in the population) and the evenness (the relative abundance of each species present), the Native (53.26) and Pad (49.11) samples had the highest biodiversity, while Flock 9 had the lowest (7.41) biodiversity (indices at 0.03). The Shannon index accounts for both abundance and evenness, and quantifies entropy or uncertainty associated with prediction of the next randomly chosen entity. Again, the Native (4.54) and Pad (4.43) samples had the highest entropy representing more diverse communities, while Flock 9 had the lowest (2.64) index (indices at 0.03). The Shannon evenness index quantifies how numerically equal community members are represented. The Native (0.88) and Pad (0.88) samples had more asymmetrical communities with some species dominating, whereas Flock 9 had the lowest (0.62) index (indices at 0.03).

**TABLE 1:** The coverage, diversity and evenness indices at 0.03 and 0.10 genetic distances of soil bacteria associated with preconstruction and subsequent flock rotations.

**Flocks**	**Coverage**		**Inverse Simpson Index**		**Shannon Index**		**Shannon Evenness Index**	
	**0.03**	**0.10**	**0.03**	**0.10**	**0.03**	**0.10**	**0.03**	**0.10**
Native	76	86	53.26	33.32	4.54	4.12	0.88	0.85
Pad	82	88	49.11	31.72	4.43	4.01	0.88	0.84
Flock 1	93	96	10.62	7.18	2.94	2.45	0.71	0.66
Flock 2	92	96	14.23	8.51	3.30	2.68	0.76	0.69
Flock 4	92	95	10.22	7.40	2.91	2.47	0.68	0.64
Flock 5	92	95	14.13	10.66	3.26	2.84	0.75	0.73
Flock 6	92	96	9.21	7.37	2.82	2.47	0.67	0.65
Flock 7	91	95	10.06	8.42	2.91	2.56	0.68	0.66
Flock 8	91	95	11.92	10.00	3.10	2.77	0.71	0.70
Flock 9	92	95	7.41	5.87	2.64	2.28	0.62	0.59
Flock 10	86	92	14.37	10.34	3.47	2.96	0.74	0.69
Flock 11	88	92	9.60	7.13	3.27	2.78	0.71	0.66

**FIGURE 2:**
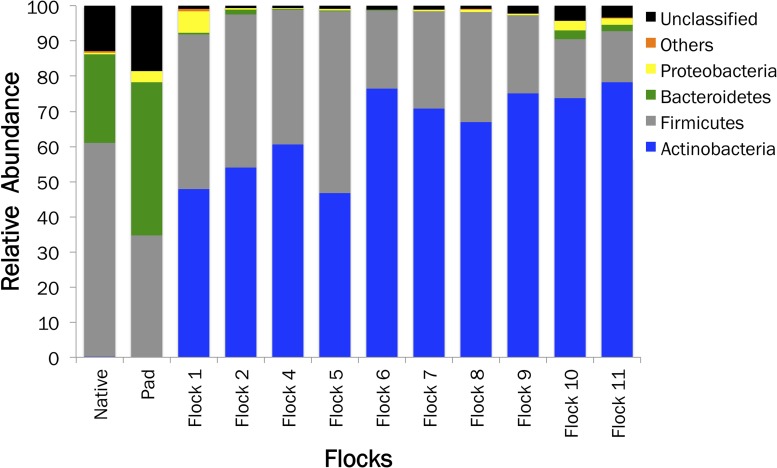
A histogram showing the relative abundances of bacterial phyla associated with Native, Pad, and Flock samples.

The relative sequence abundance in relation to the soil sample type is shown in Figure 2. An immediate change in the proportion of Bacteroidetes upon the addition of bedding and poults to the house occurred. The proportion of Actinobacteria populations increased to basically replace Bacteroidetes, while Firmicutes remained one of the dominant phyla. Additionally, a high proportion of bacteria fall into the unclassified realm from the Native and soil samples, indicating the possibility of more novel species of bacteria.

**FIGURE 3:**
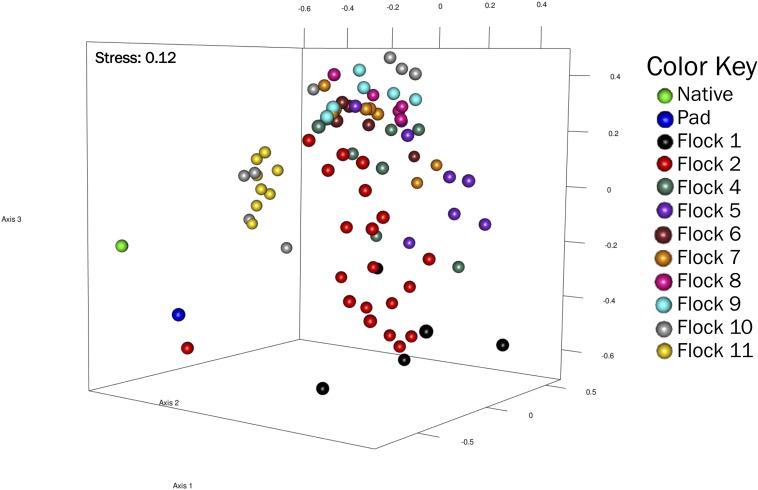
A non-metric multidimensional scaling (NMDS) plot of OTU (at 0.03 genetic distance) based on Yue and Clayton measure of dissimilarity showing clustering of Native, Pad, and Flock samples.

Non-metric multidimensional scaling (NMDS) represent a pairwise dissimilarity between objects using the theta-yc distance, in a low-dimensional space based on rank; therefore, the magnitude of distances between sequences is lost. This plot showed distinct differences (R^2^ = 0.92) in the bacterial community structures among the soils of Native, Pad and subsequent Flock samples, with Flocks 1, 2, 10, and 11 separating from Flocks 3–9 (Figure 3).

Indicator species reflect the relationship between species occurrence or abundance and the separation of samples into site groups, in this case Flock rotations. It considers the abundance of the species (specificity) and the predominance of the species (fidelity) to identify sites or perturbations within those sites. Native, Pad and Flocks 1, 2, 10, and 11 (Table 2) have indicator species for sites that cluster separately in the dendrograms. In particular, the family Lachnospiraceae, the order Clostridiales and the species *Turicibacter* and *Lactobacillus* have strong indicator values for the Native soil, along with the family Prevotellaceae for the Pad soil.

**TABLE 2:** List of Indicator bacterial taxa associated with soils collected from different flocks.

**Bacterial Group**	**Indicator Group**	**Indicator Value**	**Sequence Size**
Unclassified Lachnospiraceae	Native	100	119
Unclassified Clostridiales	Native	100	55
*Turicibacter*	Native	98	106
*Lactobacillus*	Native	93	137
*Clostridium*_sensu_stricto	Native	70	192
Unclassified bacteria	Native	59	69
*Prevotella*	Native/Pad	98	108
Unclassified Bacteriodetes	Native/Pad	90	427
Unclassified Prevotellaceae	Pad	100	100
*Brachybacterium*	Flock 1	61	1245
*Brevibacterium*	Flock 1–2	58	398
Unclassified Bacillaceae	Flock 2	51	422
Unclassified Sphingobacteriaceae	Flock 10	57	266
Unclassified Actinomycetales	Flock 10–11	65	395
*Salinicoccus*	Flock 11	61	69
*Stackebrandtia*	Flock 11	53	98

*Rare indicator taxa (OTU with less than 50 sequence reads) and indicator taxa with indictor value less than 50 are not included in this table.*

**FIGURE 4:**
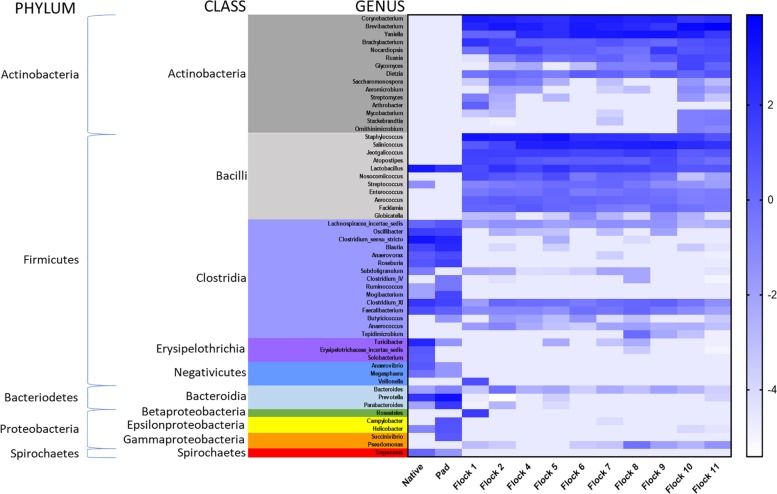
A heat map of natural log transformed quantity of genera identified sequences grouped by Class collected from Native, PAD and Flock rotation samples.

A heat map of the genus taxa level, grouped by bacterial class, show major shifts in classes between the Native and Pad samples, and subsequent Flocks (Figure 4). Due to low sample numbers AMOVAs were not run on Native and Pad, but distinct differences are seen in the heat map correlation. Results of AMOVA comparisons on the Flocks reveal that based on OTU theta-yc distance at a level of 0.03, Flocks 1 and 2 are significantly different from all other Flocks, but not from each other. Flock 4 is significantly different from Flocks 9–11 and Flock 5 from Flocks 8–11. Flock 10 is different from all, but Flock 6, and Flock 11 is significantly different from all other Flocks.

**FIGURE 5:**
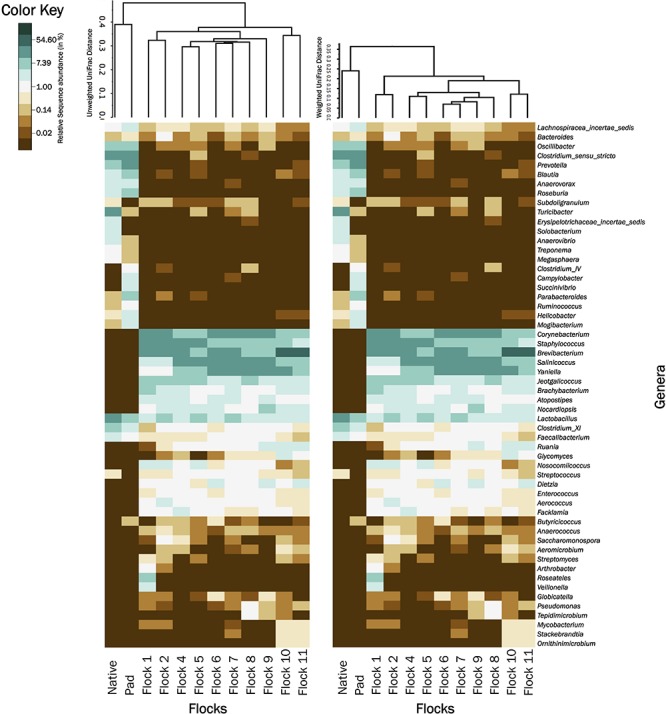
A heatmap of bacterial genera associated with Native, Pad, and Flock samples. For better visualization, relative abundance values were natural log transformed. For natural log transformation, “0” was converted into “0.01.” Samples were clustered based on unweighted **(left)** and weighted **(right)** Unifrac trees.

Figure 5 shows UPGMA tree based on weighted and unweighted Unifrac distances, respectively, and house soil microbiome heat maps at the genus level. The Native and Pad soil microbiome separate into their own clade when compared to the house soil microbiome after the introduction of bedding and birds. Further, the first two flock rotation (Flocks 1 and 2) separate together as being different from the subsequent flock rotation except for the two flock rotations that occurred after the TCO of the house (Flock 10 and 11). In the unweighted Unifrac tree, the bacterial structure associated with Flocks 4 to 9, was qualitatively more closely related to Flocks 1 and 2 than to Flocks 10 and 11. Whereas in the weighted Unifrac tree, bacteria associated with Flocks 4 to 9 was quantitatively more closely related to Flocks 10 and 11 than to Flocks 1 and 2. After the PCO (Flocks 8 and 9) only minor changes to the soil microbiome were observed.

## Discussion

The chicken gastrointestinal tract contains commensal and pathogenic bacteria, primarily obligate, facultative and aerotolerant anaerobes. It is a complex ecosystem with indigenous bacteria important for the health of the bird, but its constituents can vary with intestinal compartment, age, environmental factors, such as diet and bedding type, and the individual bird (van der Wielen et al., 2002; Crippen et al., 2008; Sheffield et al., 2009a, b; Torok et al., 2011; Pan and Yu, 2014). The composition and fluctuation of the soil microbes within a poultry production house are of special interest because of their possible impact on the health of the chickens through shaping its microbiome and of the poultry house effecting the natural soil microbiome beneath the house. Immediately after hatching, a poult begins populating its intestinal tract from bacteria acquired from its surrounding environment (van der Wielen et al., 2002). Fossil evidence of avian crops from the beginning of the early Cretaceous period, about 145.5 million years ago, demonstrates that essentially a modern avian digestive system had formed early in avian evolution (Zheng et al., 2011). These birds ate pebbles, small stones and grit to aid in the gizzard grinding process, which made up for not having teeth (Feduccia, 2011). Today, chickens typically eat small stones, pebbles or commercial grit that remain in the gizzard to aid the muscular structure in the grinding of chicken food (Jacob and Prescatore, 2013; Jacob, 2015). Therefore, one can expect that small pebbles, sand and grit will be normally ingested by the birds in the house from the soil beneath the litter, along with bacteria ingested from those sources. In this study, the Native and Pad soil had diverse microbial communities, however, after interaction with just one flock rotation the soil lost much of its diversity. Logically the soil community changes may have resulted from contact with the birds, bedding, feed, water, chicken feathers, carcasses, manure and the other elements within the house (i.e., insects, farm equipment, workers). A PCO does not usually reach the soil beneath the litter, whereas a TCO removes all of the litter plus a thin top layer of the Pad soil. The partial clean-out after Flock 7 and the full clean-out of litter after Flock 9, which were both followed by the addition of new bedding for Flocks 8 and 10, appeared to briefly increase diversity, but it again decreased substantially after one flock rotation. Thus, the working poultry house substantially diminished the microbial diversity in the soil beneath.

Ivanova et al. (2018) analyzed the microbiome community structure in various soil types and determined that it was influenced by two main factors, soil acidity, which has major influences at the phylum level, and type of vegetation, which primarily influences order family and genus. In the poultry house environment, this might correspond to pH and bedding type; in this case, pine shavings. Soil analyses for this house were done in combination with these microbiome samples and reported in a separate publication (Sheffield et al., 2015). Briefly, changes in Cu, pH, K, Mg, Mn, S, Zn, and % organic matter had the largest influence on changes within the house soil over time. Soil K, Mg, and S levels continued to increase over successive flock rotations, as did the micronutrient and heavy metals Mn, Cu, and Zn. As can be expected, % organic matter also increased with each flock rotation. The PCO had little effect on these soil parameters, but the TCO significantly reduced their levels. The pH was slightly acidic for Native and Pad in which Bacteroidetes, Firmicutes, and Proteobacteria were more prevalent. It was also slightly acidic during Flock 1 which was a mixed community that was in transition into the community found in Flocks 2–11. The pH became slightly alkaline for Flocks 2–11 in which Firmicutes and Actinobacteria were more prevalent. Alkaline pH within the house is typical of most broiler operations; even when treated with acid treatment, pH eventually returns to alkaline conditions once the birds are in the house (Elliot and Collins, 1982; Miles et al., 2013).

Nitrogen is essential for bacteria, but minimizing volatilization of ammonia (NH_3_), as well as hydrogen sulfide (H_2_S) and sulfur, is one of the difficult issues confronting the poultry producer. The increased moisture, temperature and pH within the house facilitates ammonia production and the presence of ammonia and nitrate increases when litter is reused for successive flock rotations. Most aerobic denitrifiers that convert nitrate back to nitrogen gas belong to the Proteobacteria phyla and these bacteria are important for the reduction of noxious odors emanating from the poultry facilities (Ji et al., 2015). The Gammaproteobacteria isolated from this house included *Succinivibrio* and *Pseudomonas*. *Pseudomonas aeruginosa* is probably the best studied of the pseudomonads and can cause mortality in chickens (Walker et al., 2002). Russell et al. (1995) isolated *Shewanella putrefaciens*, *Pseudomonas fluorescens*, and *P. fragi* bacteria from spoiled chicken carcasses. Pseudomonads can metabolize uric acid into ammonia and carbon dioxide, but can also act as denitrifiers (Bachrach, 1957; Ji et al., 2014). *Succinivibrio* occurred only in the Pad soil and *Pseudomonas* appeared with the birds and bedding and persisted throughout the subsequent flock rotations.

Rothrock and Locatelli (2019) stressed the need to understand the impact of the farm environment on the poultry microbiome when they found that the physical farm environment, including the soil, at two poultry farms managed differently affected the resulting OTU composition of the microbiome. Unfortunately, few such studies exist. Cressman et al. (2010) found that fresh poultry bedding of pine shavings had more bacteria of environmental origin, while used litter had more of poultry intestinal origin. They found Proteobacteria in fresh litter but not in used litter. Firmicutes were found in both, however *Lachnospiraceae* incertae sedis and *Enterococcus* predominated in bedding while intestinal microbes, *Lactobacillus*, *Staphylococcus*, *Jeotgalicoccus*, *Salinicoccus*, *Atopostipes*, and unclassified genera of the family Bacillaceae were inhabiting the used litter. This demonstrates the influence that the birds can have on the house floor environment. In our system, *Lachnospiraceae* incertae sedis and *Lactobacillus* were present in the soil before and after birds and bedding were introduced, whereas the remaining genera listed above were only present after the introduction of bedding and birds. Therefore, suggesting the possible influence of the bedding and birds on the soil in the poultry house system.

The gastrointestinal tract of the chicken has several important digestive areas. Firmicutes (70%), Bacteroidetes (12.3%) and Proteobacteria (9.3%) dominate the chicken cecum; while the intestine consists primarily of the Bacteroidetes (Qu et al., 2008; Wei et al., 2013). In our poultry house, Firmicutes and Bacteriodetes initially dominated the soil, but when bedding and poultry were added to the house, the relative abundance of soil bacteria at the phylum level showed an immediate decrease in the proportion of Bacteroidetes. Within one flock rotation Firmicutes and Actinobacteria became prominent in the soil, and this bacterial dynamic was maintained throughout the subsequent flock rotations and the partial clean-out. Actinobacteria are normally found in the soil and they have great economic importance, because agriculture and forests depend on their contributions to soil systems (Ventura et al., 2007). The Actinobacteria in the house included *Streptomyces* spp., appearing after the bedding and birds were introduced, which produce secondary metabolites notable for antibiotic qualities. Two other genera, *Ornithinimicrobium* and *Stackebrandtia* utilize nitrate, but they did not appear until after the total clean-out of litter (Groth et al., 2001; Mayilraj et al., 2006; Liu et al., 2008; Munk et al., 2009).

The Firmicutes divided into two major groups: the anaerobic Clostridia that function in the fermentation of carbohydrates producing short chained fatty acids; and the diverse Bacilli, which are obligate or facultative aerobes (Madigan et al., 2012). An interesting observation is currently being explored regarding the balance of Firmicutes and Bacteriodetes in the gut that correlates with weight gain (Ley et al., 2006; Turnbaugh et al., 2006). From the Bacilli class, only *Streptococcus* (present in Native soil) and *Lactobacillus* (present in both Native and Pad soil) were found throughout the study. All other Bacilli appeared with the introduction of the bedding and the birds. Rehman et al. (2007) reported that *Streptococci* and *Enterobacteria* spp. colonized the caeca and then the entire intestinal tract within 24 after hatching. *Streptococcus* species can be commensal in the intestinal tract of the birds, but can also cause opportunistic acute and chronic infections in poultry, such as septicemia, peritonitis, cellulitis, salpingitis and endocarditis (Jortner and Helmboldt, 1971; Sekizaki et al., 2008). So, the presence of this bacteria is beneficial as a commensal organism and should only be an issue if the birds become stressed and/or immunocompromised. *Lactobacillus* spp. also dominate the crop, the proventriculus, and the low pH environment of the ventriculus (gizzard) (Rehman et al., 2007; Yeoman et al., 2012). *Lactobacillus* have the ability to produce lactic acid and are common commensals within the digestive, and urogenital systems of chickens, but have also been isolated from soil (Chen et al., 2004); as was found in our system. Lactobacilli are used as a probiotic in chickens and have the ability to improve production and limit foodborne pathogens, and some *Lactobacillus* species will also modulate the immune responses of chickens (Mountzouris et al., 2007, 2009, 2010; Brisbin et al., 2011; De Cesare et al., 2017).

The Native and Pad soils had a higher prevalence of Bacteroidia, Clostridia, Epsilonproteobacteria, Erysipelotrichia, Negativicutes, and Spirochaetes. Most of which decreased or disappeared after the bedding and birds where introduced. Wei et al. (2013) determined that the predominant genera from the chicken cecum and intestine were *Clostridium*, *Ruminococcus*, *Lactobacillus*, and *Bacteroides*. All of these important genera were found in the Native and Pad soil prior to placement of the poults and persisted in the soil throughout flock rotations, along with *Lachnispiracea_incertae_sedis*, *Faecalibacterium*, and *Butyricicoccus.* In poultry, *Bacteroides* are a substantial anaerobic component of the gastrointestinal tract conducting carbohydrate fermentation on simple and complex sugars and polysaccharides, producing volatile fatty acids. Those fatty acids reabsorb through the large intestine, and are utilized by both the host, and other gut bacteria, to provide a large proportion of their energy requirement. While they generally participate as beneficial commensal organisms, this genus also includes species that are significant pathogens that produce potent virulence factors, and as a whole, the genus has the most antibiotic resistance mechanisms of anaerobic species (Xu and Gordon, 2003; Wexler, 2007). *Faecalibacterium* is involved in glucose fermentation, but more importantly butyrate production and anti-inflammatory affects (Sokol et al., 2008; Miquel et al., 2013). Butyrate production plays a major role in protection against pathogen invasion by modulation of the immune system (Macfarlane and Macfarlane, 2011; Ploger et al., 2012). The *Lachnospiraceae* can breakdown carbohydrates into short-chain fatty acids; a shortage of which can cause intestinal barrier dysfunction (Wong et al., 2006; Duncan et al., 2007).

Clostridia are quite versatile metabolically; able to degrade a wide range of organic materials, such as carbohydrates, organic acids, alcohols, aromatic compounds, peptides, amino acids, amines, purines and pyrimidines (Popoff and Bouvet, 2013). They inhabit the soil and the gastrointestinal tract, but many are pathogenic causing tetanus, gas gangrene, botulism and colitis (Hatheway, 1990; Popoff, 2015). Our study found *Clostridium*_sensu_stricto, IV and XI in the broiler house. Cluster IV bacteria are a large component of the commensal gut community, but under unfavorable conditions, can be players in dysbiosis (Lopetuso et al., 2013). Bacteria from *Clostridium* IV were found in the Pad soil and again in Flock 8, just after the PCO. Cluster XI bacteria includes *Clostridium difficile*, which is an important cause of nosocomial diarrhea associated substantial morbidity and mortality; and the pathogens *C. chauvoei* and *C. sordellii*, both causative agents of gangrene. Gangrenous dermatitis in poultry is usually caused by body contact with *C. septicum*, *C. perfringens* and *Staphylococcus aureus*, either singly or in combination. In this poultry house, *Staphylococcus* was prevalent during the flock rotations but not in the Native or Pad soil, whereas Cluster XI bacteria were the densest in the initial Native and Pad samples and then found throughout the flock rotations. *Butyricicoccus* was present in the Pad soil and its abundance fluctuated throughout the Flock rotations. Eeckhaut et al. (2016) utilized *Butyricicoccus pullicaecorum* as a probiotic in broilers, where it reduced body weight, but also significantly lowered feed conversion ratios. Additionally, it decreased the abundance of pathogens: *Campylobacter*, *Enterococcus* and *Escherichia/Shigella* spp., and contributed to the prevention of necrotic lesions in the birds. Therefore, its presence in the soil could be of benefit to the flock. In the house, *Campylobacter* were present (sequence identity 0.03) in the Pad soil, but did not colonize to any extent after the birds and bedding was brought in and *Enterococcus* was not found in the Pad, but was present once the birds and bedding were placed.

Antibiotic-resistant bacteria pose a considerable threat to society; therefore, having a global perspective of where these bacteria thrive and how they spread is imperative. The World Health Organization (WHO) recently published a priority list of the top 25 most likely bacteria to develop antibiotic-resistance (Tacconelli et al., 2018). In comparing that list to this system: *Pseudomonas, Enterococcus*, and *Staphylococcus* were not originally present in the soil or pad, but appeared with the introduction of bedding and birds; *Helicobacter*, which is of significance as it is also associated with enteritis and vibrionic hepatitis in poultry, was present in both the Native and Pad soil, but decreased with the introduction of bedding and birds until it reappeared after the total clean-out.

## Conclusion

The major shifts in the soil microbiome related to the introduction of bird and bedding onto the soil, and the TCO. Actinobacteria and Bacilli were not well represented in the soil prior to the introduction of bedding and birds, but were the largest classes of soil bacteria after bedding and birds were placed and during the subsequent flock rotations. Actinobacteria contain aerobic species capable of producing phosphatase and reducing nitrates which are always a concern of producers. Bacteriodia, Erysipelotrichia, Negativicutes, Spriochetes, and Beta-, Gamma-, and Epsilonproteobacteria generally decreased in soil population after the introduction of bedding and birds. Members of the class Clostridia varied in their response to the addition of the broiler house onto Native and Pad soil. In general, few of the bacteria found in the Native and Pad soils appeared to survive in abundance after bedding and birds were added. *Lactobacillus*, *Bacteroidetes*, *Lachnospiracea*, *Clostridium_XI*, and *Faecalibacterium* were the exceptions and remained consistent community members throughout the study. The PCO did not change the soil bacterial community structure in any substantial way, only spurring a temporary increase in the populations of *Subdoligranulum*, *Clostridium_XI*, *Tepidmicrobium*, and *Pseudomonas*. Conversely, the TCO had a larger effect on the soil community composition, spurring an increase in cohort of Actinobacteria. Overall, the soil bacterial community structure was significantly affected by the addition of birds and bedding into the house and was also affected, but to a lesser extent, by clean-out management practices during the production of broilers. Thereby, the scheduling of clean-out procedures should be to maximize advantageous bacterial community structure for interaction of poults and adults.

## Data Availability

The datasets generated for this study can be found in the European Nucleotide Archive Database as part of the study PRJEB29406 (accession # ERS2859789–ERS2859880).

## Author Contributions

TC and CS conceived of the idea, designed the study, performed the experiments and analyses, and wrote the manuscript. BS and TC performed the bioinformatics and graphical presentations. JB participated in the sample collection. TC, CS, BS, and RB reviewed and edited the final manuscript.

## Conflict of Interest Statement

The authors declare that the research was conducted in the absence of any commercial or financial relationships that could be construed as a potential conflict of interest.

## Footnotes

1 http://www.researchandtesting.com/

2 http://tree.bio.ed.ac.uk/

## References

[B1] Bachrach U. (1957). The aerobic breakdown of uric acid by certain pseudomonads. *J. Gen. Microbiol.* 17 1–11. 10.1099/00221287-17-1-1 13475666

[B2] Bakker M. G. Looft T. Alt D. P. Delate K. Cambardella C. A. (2018). Bulk soil bacterial community structure and function respond to long-term organic and conventional agricultural management. *Can. J. Microbiol.* 64 901–914. 10.1139/cjm-2018-0134 30058369

[B3] Brisbin J. T. Gong J. Orouji S. Esufali J. Mallick A. I. Parvizi P. (2011). Oral treatment of chickens with lactobacilli influences elicitation of immune responses. *Clin. Vaccine Immunol.* 18 1447–1455. 10.1128/CVI.05100-11 21734067 PMC3165221

[B4] Chen Y.-S. Yanagida F. Shinohara T. (2004). Isolation and identification of lactic acid bacteria from soil using an enrichment procedure. *Lett. Appl. Microbiol.* 40 195–200. 10.1111/j.1472-765x.2005.01653.x 15715644

[B5] Cressman M. D. Yu Z. Nelson M. C. Moeller S. J. Lilburn M. S. Zerby H. N. (2010). Interrelations between the Microbiotas in the Litter and in the Intestines of Commercial Broiler Chickens. *Appl. Environ. Microbiol.* 76 6572–6582. 10.1128/aem.00180-10 20693454 PMC2950482

[B6] Crippen T. L. Sheffield C. L. Andrews K. Dowd S. E. Bongaerts R. J. Nisbet D. J. (2008). Planktonic and biofilm community characterization and *Salmonella* resistance of 14-day-old chicken cecal microflora-derived continuous-flow cultures. *J. Food Prot.* 71 1981–1987. 10.4315/0362-028x-71.10.1981 18939741

[B7] De Cesare A. Sirri F. Manfreda G. Moniaci P. Giardini A. Zampiga M. (2017). Effect of dietary supplementation with *Lactobacillus acidophilus* d2/csl (cect 4529) on caecum microbioma and productive performance in broiler chickens. *PLoS One* 12:e0176309. 10.1371/journal.pone.0176309 28472118 PMC5417446

[B8] Dowd S. E. Crippen T. L. Sun Y. Gontcharova V. Youn E. Muthaiyan A. (2010). Microarray analysis and draft genomes of two *Escherichia coli* O157:H7 lineage ii cattle isolates FRIK966 and FRIK2000 investigating lack of shiga toxin expression. *Foodborne Pathog. Dis.* 7 763–773. 10.1089/fpd.2009.0482 20156085

[B9] Duncan S. H. Louis P. Flint H. J. (2007). Cultivatable bacterial diversity from the human colon. *Lett. Appl. Microbio.* 44 343–350. 10.1111/j.1472-765x.2007.02129.x 17397470

[B10] Edgar R. C. Haas B. J. Clemente J. C. Quince C. Knight R. (2011). Uchime improves sensitivity and speed of chimera detection. *Bioinformatics* 27 2194–2200. 10.1093/bioinformatics/btr381 21700674 PMC3150044

[B11] Eeckhaut V. Wang J. Van Parys A. Haesebrouck F. Joossens M. Falony G. (2016). The probiotic butyricicoccus pullicaecorum reduces feed conversion and protects from potentially harmful intestinal microorganisms and necrotic enteritis in broilers. *Front. Microbiol.* 7:1416. 10.3389/fmicb.2016.01416 27708624 PMC5030265

[B12] Elliot H. A. Collins N. E. (1982). Factors affecting ammonia release in broiler houses. *Trans ASAE* 25 413–424.

[B13] Fadiel A. Anidi I. Eichenbaum K. D. (2005). Farm animal genomics and informatics: an update. *Nucleic Acids Res.* 33 6308–6318. 10.1093/nar/gki931 16275782 PMC1278942

[B14] Feduccia A. (2011). Cretaceous avian crops reveal dietary secrets and pose evolutionary questions. *PNAS* 108 16487–16488. 10.1073/pnas.1113314108 21949395 PMC3189070

[B15] Godfray H. C. Beddington J. R. Crute I. R. Haddad L. Lawrence D. Muir J. F. (2010). Food security: the challenge of feeding 9 billion people. *Science* 327 812–818. 10.1126/science.1185383 20110467

[B16] Gould F. Hoffman G. Rechenthin C. (1960). *Vegetational Areas of Texas.* College Station, TX: Texas A&M Engineering Texas Experiment Station.

[B17] Groth I. Schumann P. Weiss N. Schuetze B. Augsten K. Stackebrandt E. (2001). Ornithinimicrobium humiphilum gen. nov., sp. nov., a novel soil actinomycete with L-ornithine in the peptidoglycan. *Int. J. Syst. Evol. Microbiol.* 51 81–87. 10.1099/00207713-51-1-81 11211277

[B18] Hatheway C. L. (1990). Toxigenic clostridia. *Clin. Microbiol. Rev.* 3 66–98. 10.1128/cmr.3.1.66 2404569 PMC358141

[B19] Ivanova E. A. Pershina E. V. Kutovaya O. V. Sergalieva N. K. Nagieva A. G. Zhiengaliev A. T. (2018). Comparative analysis of microbial communities of contrasting soil types in different plant communities. *Russian J. Ecol.* 49 30–39. 10.1134/s106741361801006x

[B20] Jacob J. (2015). *Avian Digestive System: SMALL and Backyard Flocks.* Available at: https://articles.extension.org/pages/65376/avian-digestive-system (accessed May 23, 2019).

[B21] Jacob J. Prescatore T. (2013). *Avian Digestive System.* Available at: http://www2.ca.uky.edu/agcomm/pubs/asc/asc203/asc203.pdf (accessed May 23, 2019).

[B22] Ji B. Wang H. Yang K. (2014). Tolerance of an aerobic denitrifier (*Pseudomonas stutzeri*) to high O_2_ concentrations. *Biotechnol. Lett.* 36 719–722. 10.1007/s10529-013-1417-x 24347061

[B23] Ji B. Yang K. Zhu L. Jiang Y. Wang H. Zhou J. (2015). Aerobic denitrification: a review of important advances of the last 30 years. *Biotechnol. Bioprocess Eng.* 20 643–651. 10.1007/s12257-015-0009-0

[B24] Jortner B. S. Helmboldt C. F. (1971). Streptococcal bacterial endocarditis in chickens. associated lesions of the central nervous system. *Vet. Pathol.* 8 54–62. 10.1177/030098587100800107 5003378

[B25] Ley R. E. Turnbaugh P. J. Klein S. Gordon J. I. (2006). Microbial ecology: human gut microbes associated with obesity. *Nature* 444 1022–1023. 17183309 10.1038/4441022a

[B26] Liu X. Y. Wang B. J. Jiang C. Y. Liu S. J. (2008). Ornithinimicrobium pekingense sp. nov., isolated from activated sludge. *Int. J. Syst. Evol. Microbiol.* 58 116–119. 10.1099/ijs.0.65229-0 18175694

[B27] Lopetuso L. R. Scaldaferri F. Petito V. Gasbarrini A. (2013). Commensal clostridia: leading players in the maintenance of gut homeostasis. *Gut Pathog.* 5:23. 10.1186/1757-4749-5-23 23941657 PMC3751348

[B28] Macfarlane G. T. Macfarlane S. (2011). Fermentation in the human large intestine: its physiologic consequences and the potential contribution of prebiotics. *J. Clin. Gastroenterol.* 45(Suppl), S120–S127. 10.1097/MCG.0b013e31822fecfe 21992950

[B29] Madigan M. Martinko J. Stahl D. Clark D. (2012). *Brock Biology of Microorganisms Thirteenth.* San Francisco, CA: Pearson Education Inc.

[B30] Mathews K. Haley M. (2014). *Pork and Poultry Offset Declines in Beef Production.* Washington, DC: U.S. Department of Agriculture.

[B31] Mayilraj S. Saha P. Suresh K. Saini H. S. (2006). Ornithinimicrobium kibberense sp. nov., isolated from the Indian Himalayas. *Int. J. Syst. Evol. Microbiol.* 56 1657–1661. 10.1099/ijs.0.64138-0 16825645

[B32] Miles D. Brooks J. McLaughlin M. Rowe D. (2013). Broiler litter ammonia emissions near sidewalls, feeders, and waterers. *Poult. Sci.* 92 1693–1698. 10.3382/ps.2012-02809 23776254

[B33] Miquel S. Martin R. Rossi O. Bermúdez-Humarán L. G. Chatel J. M. Sokol H. (2013). *Faecalibacterium prausnitzii* and human intestinal health. *Curr. Opin. Microbiol.* 16 255–261. 10.1016/j.mib.2013.06.003 23831042

[B34] Mountzouris K. C. Balaskas C. Xanthakos I. Tzivinikou A. Fegeros K. (2009). Effects of a multi-species probiotic on biomarkers of competitive exclusion efficacy in broilers challenged with *Salmonella enteritidis*. *Br. Poult. Sci.* 50 467–478. 10.1080/00071660903110935 19735016

[B35] Mountzouris K. C. Tsirtsikos P. Kalamara E. Nitsch S. Schatzmayr G. Fegeros K. (2007). Evaluation of the efficacy of a probiotic containing *lactobacillus*, *bifidobacterium*, *enterococcus*, and *pediococcus* strains in promoting broiler performance and modulating cecal microflora composition and metabolic activities. *Poult. Sci.* 86 309–317. 10.1093/ps/86.2.309 17234844

[B36] Mountzouris K. C. Tsitrsikos P. Palamidi I. Arvaniti A. Mohnl M. Schatzmayr G. (2010). Effects of probiotic inclusion levels in broiler nutrition on growth performance, nutrient digestibility, plasma immunoglobulins, and cecal microflora composition. *Poult. Sci.* 89 58–67. 10.3382/ps.2009-00308 20008803

[B37] Munk C. Lapidus A. Copeland A. Jando M. Mayilraj S. Glavina Del Rio T. (2009). Complete genome sequence of *Stackebrandtia nassauensis* type strain (llr-40k-21). *Stand. Genomic Sci.* 1 234–241. 10.4056/sigs.47643 21304662 PMC3035245

[B38] Pan D. Yu Z. (2014). Intestinal microbiome of poultry and its interaction with host and diet. *Gut Microbes* 5 108–119. 10.4161/gmic.26945 24256702 PMC4049927

[B39] Ploger S. Stumpff F. Penner G. B. Schulzke J. D. Gabel G. Martens H. (2012). Microbial butyrate and its role for barrier function in the gastrointestinal tract. *Ann. N. Y. Acad. Sci.* 1258 52–59. 10.1111/j.1749-6632.2012.06553.x 22731715

[B40] Popoff M. R. (2015). From saprophytic to toxigenic clostridia, a complex evolution based on multiple diverse genetic transfers and/or rearrangements. *Res. Microbiol.* 166 221–224. 10.1016/j.resmic.2015.02.008 25744779

[B41] Popoff M. R. Bouvet P. (2013). Genetic characteristics of toxigenic clostridia and toxin gene evolution. *Toxicon* 75 63–89. 10.1016/j.toxicon.2013.05.003 23707611

[B42] Qu A. Brulc J. M. Wilson M. K. Law B. F. Theoret J. R. Joens L. A. (2008). Comparative metagenomics reveals host specific metavirulomes and horizontal gene transfer elements in the chicken cecum microbiome. *PLoS One* 3:e2945. 10.1371/journal.pone.0002945 18698407 PMC2492807

[B43] Quince C. Lanzen A. Davenport R. J. Turnbaugh P. J. (2011). Removing noise from pyrosequenced amplicons. *BMC Bioinformatics* 12:38. 10.1186/1471-2105-12-38 21276213 PMC3045300

[B44] R Development.Core.Team. (2011). *R: A Language and Environment for Statistical Computing.* Vienna: R Foundation for Statistical Computing.

[B45] Rehman H. U. Vahjen W. Awad W. A. Zentek J. (2007). Indigenous bacteria and bacterial metabolic products in the gastrointestinal tract of broiler chickens. *Arch. Anim. Nutr.* 61 319–335. 10.1080/17450390701556817 18030916

[B46] Rothrock J. R. Locatelli A. (2019). Importance of farm environment to shape poultry-related microbiomes throughout the farm-to-fork continuum of pasture-raised broiler flocks. *Front. Sust. Food. Syst.* 3 1–15. 10.3389/fvets.2019.00157 31179291 PMC6543280

[B47] Russell S. M. Fletcher D. L. Cox N. A. (1995). Spoilage bacteria of fresh broiler chicken carcasses. *Poult. Sci.* 74 2041–2047. 10.3382/ps.0742041 8825595

[B48] Schloss P. D. Westcott S. L. Ryabin T. Hall J. R. Hartmann M. Hollister E. B. (2009). Introducing mothur: open-source, platform-independent, community-supported software for describing and comparing microbial communities. *Appl. Environ. Microbiol.* 75 7537–7541. 10.1128/AEM.01541-09 19801464 PMC2786419

[B49] Sekizaki T. Nishiya H. Nakajima S. Nishizono M. Kawano M. Okura M. (2008). Endocarditis in chickens caused by subclinical infection of *Streptococcus gallolyticus* subsp. *gallolyticus*. *Avian Dis.* 52 183–186. 10.1637/8048-070307-case 18459321

[B50] Shange R. S. Ankumah R. O. Zabawa R. Dowd S. E. (2013). Bacterial community structure and composition in soils under industrial poultry production activities: an observational study. *Air Soil Water Res.* 6 91–101.

[B51] Sheffield C. L. Crippen T. L. Andrews K. Bongaerts R. J. Nisbet D. J. (2009a). Characterization of planktonic and biofilm communities of day-of-hatch chicks cecal microflora and their resistance to *Salmonella* colonization. *J. Food Prot.* 72 959–965. 10.4315/0362-028x-72.5.959 19517721

[B52] Sheffield C. L. Crippen T. L. Andrews K. Bongaerts R. J. Nisbet D. J. (2009b). Planktonic and biofilm communities from 7-day-old chicken cecal microflora cultures: characterization and resistance to *Salmonella* colonization. *J. Food Prot.* 72 1812–1820. 10.4315/0362-028x-72.9.1812 19777880

[B53] Sheffield C. L. Crippen T. L. Byrd J. A. Beier R. C. Yeater K. (2015). Canonical discrimination of the effect of a new broiler production facility on soil chemical profiles as related to current management practices. *PLoS One* 10:e0128179. 10.1371/journal.pone.0128179 26029909 PMC4452585

[B54] Sokol H. Pigneur B. Watterlot L. Lakhdari O. Bermúdez-Humarán L. G. Gratadoux J. J. (2008). *Faecalibacterium prausnitzii* is an anti-inflammatory commensal bacterium identified by gut microbiota analysis of Crohn disease patients. *Proc. Natl. Acad. Sci. U. S. A.* 105 16731–16736. 10.1073/pnas.0804812105 18936492 PMC2575488

[B55] Tacconelli E. Carrara E. Savoldi A. Harbarth S. Mendelson M. Monnet D. L. (2018). Discovery, research, and development of new antibiotics: the who priority list of antibiotic-resistant bacteria and tuberculosis. *Lancet Infect. Dis.* 18 318–327. 10.1016/S1473-3099(17)30753-3 29276051

[B56] Torok V. A. Hughes R. J. Mikkelsen L. L. Perez-Maldonado R. Balding K. MacAlpine R. (2011). Identification and characterization of potential performance-related gut microbiotas in broiler chickens across various feeding trials. *Appl. Environ. Microbiol.* 77 5868–5878. 10.1128/AEM.00165-11 21742925 PMC3165380

[B57] Turnbaugh P. J. Ley R. E. Mahowald M. A. Magrini V. Mardis E. R. Gordon J. I. (2006). An obesity-associated gut microbiome with increased capacity for energy harvest. *Nature* 444 1027–1031. 17183312 10.1038/nature05414

[B58] Usda. (2018a). *Broilers: Production by Year, us.* Washington, DC: U.S. Department of Agriculture.

[B59] Usda. (2018b). *Livestock and Poultry: World Markets and Trade.* Washington, DC: United States Department of Agriculture.

[B60] van der Wielen P. W. Keuzenkamp D. A. Lipman L. J. van Knapen F. Biesterveld S. (2002). Spatial and temporal variation of the intestinal bacterial community in commercially raised broiler chickens during growth. *Microb. Ecol.* 44 286–293. 10.1007/s00248-002-2015-y 12219265

[B61] Ventura M. Canchaya C. Tauch A. Chandra G. Fitzgerald G. F. Chater K. F. (2007). Genomics of actinobacteria: tracing the evolutionary history of an ancient phylum. *Microbiol. Mol. Biol. Rev.* 71 495–548. 10.1128/mmbr.00005-07 17804669 PMC2168647

[B62] Walker S. E. Sander J. E. Cline J. L. Helton J. S. (2002). Characterization of *Pseudomonas aeruginosa* isolates associated with mortality in broiler chicks. *Avian. Dis.* 46 1045–1050. 10.1637/0005-2086(2002)046 12495073

[B63] Wang Q. Garrity G. M. Tiedje J. M. Cole J. R. (2007). Naïve bayesian classifier for rapid assignment of rRNA sequences into the new bacterial taxonomy. *Appl. Environ. Microbiol.* 73 5261–5267. 10.1128/aem.00062-07 17586664 PMC1950982

[B64] Wei S. Morrison M. Yu Z. (2013). Bacterial census of poultry intestinal microbiome. *Poult. Sci.* 92 671–683. 10.3382/ps.2012-02822 23436518

[B65] Wexler H. M. (2007). *Bacteroides*: the good, the bad, and the nitty-gritty. *Clin. Microbiol. Rev.* 20 593–621. 10.1128/cmr.00008-07 17934076 PMC2176045

[B66] Wong J. M. de Souza R. Kendall C. W. Emam A. Jenkins D. J. (2006). Colonic health: fermentation and short chain fatty acids. *J. Clin. Gastroenterol.* 40 235–243. 16633129 10.1097/00004836-200603000-00015

[B67] Xu J. Gordon J. I. (2003). Honor thy symbionts. *Proc. Natl. Acad. Sci. U.S.A.* 100 10452–10459. 10.1073/pnas.1734063100 12923294 PMC193582

[B68] Yeoman C. J. Chia N. Jeraldo P. Sipos M. Goldenfeld N. D. White B. A. (2012). The microbiome of the chicken gastrointestinal tract. *Anim. Health Res. Rev.* 13 89–99. 10.1017/S1466252312000138 22853945

[B69] Zheng X. Martin L. D. Zhou Z. Burnham D. A. Zhang F. Miao D. (2011). Fossil evidence of avian crops from the early cretaceous of china. *PNAS* 108 15904–15907. 10.1073/pnas.1112694108 21896733 PMC3179114

