# Supplementary material for: How Management Practices Within a Poultry House During Successive Flock Rotations Change the Structure of the Soil Microbiome
Source: Front Microbiol. 2019 Sep 13;10:2100. doi: 10.3389/fmicb.2019.02100 (PMC6753631; doi:10.3389/fmicb.2019.02100)
Supplement: Supplementary file 1 [file Table_1.pdf]

Supplemental Table 1. The feeding regime and basic composition of the five ration types corresponding to bird age fed to the poultry over the grow-out period during one flock rotation. Note detailed feed composition is proprietary.

**Bird Age (days)**	**Ration**	**Crude Protein (%)**	**Vitamin (%)**	**Trace Mineral (%)**	**Metabolizable Energy (Kcal Kg^{-1})**
1-14	Starter	20	100	100	2850
15-28	Grower	19	80	80	2900
29-39	Finisher #1	16	60	60	3000
40-46	Finisher #2[a]	15	40	40	3000
47-50	Withdrawal[b]	15	40	40	3000

[a] If birds are older than 50 days, Finisher #2 is fed for a longer period of time

[b] Withdrawal ration is fed only for the last 3 days of grow-out, and it has the same composition as Finisher #2 without additives.
